# A multi-channel uncertainty-aware multi-resolution network for MR to CT synthesis

**DOI:** 10.3390/app11041667

**Published:** 2021-02-12

**Authors:** Kerstin Kläser, Pedro Borges, Richard Shaw, Marta Ranzini, Marc Modat, David Atkinson, Kris Thielemans, Brian Hutton, Vicky Goh, Gary Cook, M Jorge Cardoso, Sébastien Ourselin

**Affiliations:** 1 Dept. Medical Physics & Biomedical Engineering, University College London, UK; 2 School of Biomedical Engineering & Imaging Sciences, King’s College London, UK; 3 Institute of Nuclear Medicine, University College London, UK; 4 Centre for Medical Imaging, University College London, UK

**Keywords:** MR to CT synthesis, Multi-resolution CNN, Uncertainty

## Abstract

Synthesising computed tomography (CT) images from magnetic resonance images (MRI) plays an important role in the field of medical image analysis, both for quantification and diagnostic purposes. Convolutional neural networks (CNNs) have achieved state-of-the-art results in image-to-image translation for brain applications. However, synthesising whole-body images remains largely uncharted territory involving many challenges, including large image size and limited field of view, complex spatial context, and anatomical differences between images acquired at different times. We propose the use of an uncertainty-aware multi-channel multi-resolution 3D cascade network specifically aiming for whole-body MR to CT synthesis. The Mean Absolute Error on the synthetic CT generated with the MultiRes_*unc*_ network (73.90 HU) is compared to multiple baseline CNNs like 3D U-Net (92.89 HU), HighRes3DNet (89.05 HU) and deep boosted regression (77.58 HU) and shows superior synthesis performance. We ultimately exploit the extrapolation properties of the MultiRes networks on sub-regions of the body.

## 1 Introduction

Simultaneous positron emission tomography and magnetic resonance imaging (PET/MRI) is an important tool in both clinical and research applications, allowing a multiparametric evaluation of the subject. MRI provides information with high soft-tissue contrast and PET gives information about the radiotracer uptake distribution. For the PET reconstruction process, it is essential to perform photon attenuation correction (AC) throughout the subject. A multi-center study on brain images has shown that obtaining tissue attenuation coefficients from synthesised computed tomography (CT) images leads to state-of-the-art results for PET/MRI AC [1]. Within the last years, many research groups have utilised convolutional neural networks (CNNs) in the field of MR to CT synthesis that have proved to be a powerful tool in the image-to-image translation task, outperforming existing multi-atlas-based methods [2,3]. However, little progress has been made in the area of whole-body MR to CT synthesis. The biggest hurdle with high-resolution whole-body data is its size and the fact that a large field of view is crucial for making accurate pseudo-CT (pCT) predictions. In 2019, Ge et al. [4] attempted to translate whole-body MR images to CT images by introducing a multi-view adversarial learning scheme that predicts 2D pCT images along three axes (axial, coronal, sagittal). They obtain 3D volumes for each axis by stacking 2D slices together, followed by an average fusion that results in the final 3D volume. The synthesis performance is evaluated on multiple sub-regions of the body (lungs, femur bones, spine etc.), however, the authors do not provide results on the full volume.

Learning image features from multiple levels of resolution has been used for many computer vision tasks including dynamic scene deblurring [5], optical flow prediction [6] and depth map estimation [7]. In the field of medical imaging, multi-resolution learning has been utilised to solve image classification [8], super-resolution [9] and segmentation [10] tasks. These methods learn strong features at multiple levels of scale and abstraction, therefore finding the input/output voxel correspondence based on these features. The large image size of whole-body images and GPU memory constraints do not allow for high-resolution 3D image synthesis networks to be trained on full images. Thus, training is performed in a patch-wise manner, which only captures a limited amount of spatial context.

Modelling uncertainty has gained popularity in the field of medical imaging, specifically in areas like image translation, segmentation, and super-resolution [11–13] and still remains an active field of research. It is important to distinguish between two types of uncertainty: aleatoric and epistemic uncertainty [14]. Aleatoric uncertainty captures the irreducible variance that exists in the data, whereas epistemic uncertainty accounts for the uncertainty in the model [15]. Aleatoric uncertainty can be further divided into two subcategories: homoscedastic and heteroscedastic uncertainty. Homoscedastic uncertainty is constant across all input data, while heteroscedastic uncertainty varies across the input data.

In this work, we propose the use of a deep learning framework for multi-resolution image translation specifically designed for whole-body MR to CT synthesis (MultiRes). We demonstrate that incorporating feature maps learned at multiple resolutions results in a significantly better synthesis result compared to using high-resolution images only. We further present the benefits afforded by incorporating model uncertainty [16] at multiple resolutions for whole-body MR to CT synthesis.

This paper is an extension of preliminary work [17]. We extend the framework to allow for multi-channel inputs and add additional validation on a brain dataset showing state-of-the-art synthesis results.

## 2 Methods

Neural networks commonly used in this field, such as the U-Net [18] with standard parameters, can only store patches of size 160^3^ on a 32GB VRAM GPU. This relatively small field of view causes significant issues as we will show in the experiments section.

We use an end-to-end multi-scale CNN to enlarge the field of view while maintaining high-resolution features. The network takes input patches from whole-body MR images at three resolution levels to synthesise high resolution, realistic CT patches.

We further incorporate uncertainty in our network. It is evident that in our setting the aleatoric uncertainty should be modelled as heteroscedastic, as task performance is expected to vary spatially due to the presence of artefacts, tissue boundaries, and small structures. Thus, the network incorporates explicit heteroscedastic uncertainty modelling by casting our task likelihood probabilistically. Epistemic uncertainty estimation is performed via traditional Monte Carlo dropout. More specifically, the network is trained with channel dropout enabling us to stochastically sample from the approximate posterior over the network weights to obtain epistemic uncertainty measures [19]. The network is encouraged to assign high levels of uncertainty to regions with large residual errors, providing a means of understanding what aspects of the data pose the greatest challenges.

**Figure 1:**
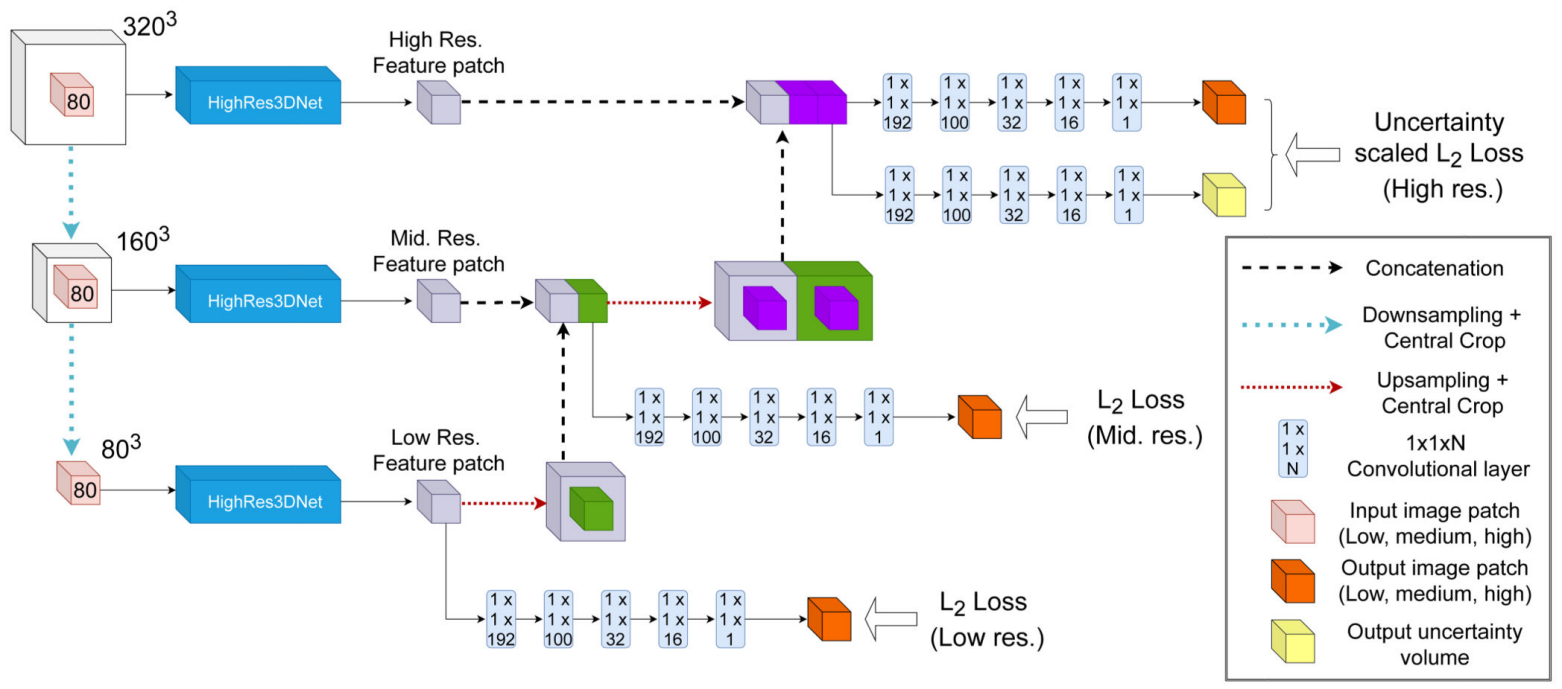
MultiRes_*unc*_ network architecture. *T*_1_ and *T*_2_ MR patches from the same subject are fed into each independent instance of the HighRes3DNet architecture [20] at various levels of resolution and field of view. A downsampling operation followed by a central crop at every level ensures the patch size is maintained while obtaining patches with different fields of view and resolution. Lower level feature maps are concatenated to those at the next level until the full resolution level, where these concatenated feature maps are passed through two branches consisting of a series of 1 × 1 × N convolutional layers: one resulting in a high-resolution synthesised pCT patch and the other in the corresponding voxel-wise heteroscedastic uncertainty.

We employ a patch-based training approach whereby at each resolution level of the framework a combination of downsampling and cropping operations results in patches of similar size but at different resolutions, spanning varied fields of view. Three independent instances of HighRes3DNet are trained simultaneously with multi-channel input, not sharing weights, taking patches of each resolution as input each resulting in a feature map with different resolution. Lower level feature maps are concatenated to those at the next level of resolution until the full resolution level, where these concatenated feature maps are passed through two branches of 1 × 1 × N convolutional layers resulting in a synthesised CT patch and the corresponding voxel-wise heteroscedastic uncertainty. Figure 1 illustrates the MultiRes framework.

We posit, similarly to [10], that the multi-resolution framework allows the network to benefit from the fine details afforded by the highest resolution patch while simultaneously increasing the spatial context provided by the larger field of view of the low-resolution patches. However, compared to [10] we incorporate an additional level of deep supervision into our MultiRes network. Compared to the MultiRes network in our preliminary work, we extend the network architecture allowing us to train with multi-channel input data (here *T*_1_ and *T*_2_-weighted MR images).

### 2.1 Modelling heteroscedastic uncertainty

Previous works on MR to CT synthesis have shown that residual errors are not homogeneously distributed throughout the entire image. It can be observed that they are largely concentrated around organ/tissue boundaries. Thus, the most suitable aleatoric uncertainty model for this task is a heteroscedastic one, which assumes that the data-dependent, or intrinsic, uncertainty is variable. Firstly, we model our task likelihood as a normal distribution with mean ***f^W^(x)***, the model output corresponding to the input **x**, parameterised by weights **W**, and voxel-wise standard deviation *σ^W^(x)*, the data intrinsic noise can be described as:

(1) $$p\left(y|f^{W}(x)\right)=𝒩\left(f^{W}(x),\sigma^{W}(x)\right)$$

We then derive our loss function by calculating the negative log of the likelihood:

(2) $$\begin{array}{l} ℒ\left(y,x;\text{W}\right)=-\log p(y|f^{W}(x))\\ \;\approx \frac{1}{2\sigma^{W}{\left(x\right)}^{2}}{\left(y-f^{W}(x)\right)}^{2}+\log\sigma^{W}(x)\\ \;=\frac{1}{2\sigma^{W}(x)}{ℒ}_{2}{\left(y,f^{W}(x)\right)}^{2}+\log\sigma^{W}(x) \end{array}$$

In regions, where a high *ℒ*_2_ error is observed, the uncertainty should compensate while also increasing. The second term in the loss function prevents the network from collapsing to the trivial solution where a large uncertainty is assigned everywhere.

### 2.2 Modelling epistemic uncertainty

The most popular method to estimate model uncertainty is test-time dropout, a Bayesian approximation at inference time. When dropout is employed during training and testing it is possible to sample from a distribution of sub-nets that in the regime of data scarcity will provide varying predictions. This variability allows the network to capture the uncertainty present in its parameters and acts as a means to estimate the voxel-wise variance across these samples. Here, we use channel dropout instead of the traditional neuron dropout. Hou et al. have demonstrated that channel dropout indeed performs better for convolutional layers where channels fully encode image features while neurons individually do not encode such meaningful information [19]. At inference time, we perform N stochastic forward passes over the network, equivalent to sampling from the posterior over the weights, to acquire N dropout samples. Ultimately, the epistemic uncertainty is obtained by calculating the variance over all dropout samples on a voxel-wise basis.

### 2.3 Implementation details

We implemented all methods with NiftyNet, a TensorFlow based open-source deep learning framework specifically developed for medical imaging [21], and code will be made available on publication. The multi-scale network consists of three independent instances of HighRes3DNet, a high-resolution, compact convolutional network. Each network takes two 80 × 80 × 80 MR image patches (*T*_1_ and *T*_2_) with different resolutions and fields of view as input. In order of high, medium, and low resolution, the MR patches are obtained by taking an initial high-resolution 320 × 320 × 320 patch and cropping the central 80 × 80 × 80 region (high), downsampling the initial patch by a factor of two and taking the central 80 × 80 × 80 patch (medium), and finally downsampling the initial patch by a factor of four to obtain a 80 × 80 × 80 patch (low). This series of cropping and downsampling operations keeps the input patch size constant for each network while increasing the field of view.

The output feature maps of each network are combined in the same but reversed manner. Starting from the lowest resolution sub-net, the output feature map of size 80 × 80×80 is upsampled by a factor of two and centrally cropped. This patch is concatenated with the output feature map of the medium resolution sub-net. This concatenated patch of size 80 × 80 × 80 × 2 is then upsampled by a factor of two and centrally cropped, before being concatenated to the output feature map of the high-resolution sub-net. These upsampling and crop operations ensure that the final output feature maps contain the same field of view prior to the final set of four 3D convolutions of kernel size 1 × 1 × 1, which generates the pCT patch.

An additional series of four 1 × 1 × 1 convolutional layers, that are identical to the convolutional layers for the synthesis branch, is used to model heteroscedastic uncertainty. For both training and testing, we set the channel dropout probability (i.e. the probability to discard any one channel) to 0.5 and *N*=50 forward passes were carried out for each experiment. The batch size was set to one, the network was optimised using the ADAM optimiser and all networks were trained until convergence. Here, convergence was defined as a sub 5% change of the loss over a period of 5000 iterations.

### 2.4 Data

The whole-body dataset used for training and validation consisted of 32 pairs of *T*_1_- and *T*_2_-weighted MR images (voxel size 0.67 × 0.67 × 5 mm^3^) and CT images (voxel size 1.37 × 1.37 × 3.27 mm^3^). Both MR and CT images were acquired in consecutive sessions on the same day. Whole-body MR images were acquired in four stages, thus requiring pre-processing to generate a uniform MR image. Firstly, the bias-field within each MR image was corrected. Secondly, the four distinct stages were fused using a percentile-based intensity harmonisation approach. All images were then resampled to CT resolution. In order to align MR and CT images, a two-step registration approach was performed. In the first step, MR and CT images were aligned using a rigid registration algorithm followed by a very-low-degree-of-freedom non-rigid deformation. The second step included a non-linear registration, which used a cubic B-spline with normalised mutual information to correct for soft tissue shift [22,23].

An additional head dataset was used for training and validation on a different region of the body. The dataset consisted of pairs of *T*_1_- and *T*_2_-weighted MR (voxel size 1.1 × 1.1 × 1.1 mm^3^) and CT (voxel size 0.586 × 0.586 × 2.5 mm^3^) images of 20 patients. The data was co-registered following the same series of rigid and non-rigid registration operations as for the whole-body data. All images were then resampled to approximately 1mm-isotropic resolution.

All images within both datasets were rescaled to be between 0 and 1 as it has been found to increase stability during training.

### 2.5 Experiments

We quantitatively and qualitatively compare the synthesis results of the MultiRes network trained with *T*_1_- and *T*_2_-weighted MR input images against three baselines: HighRes3DNet trained with patches of size 96 × 96 × 96, Deep Boosted Regression (DBR) trained with patches of size 80 × 80 × 80 and U-Net trained with 3D, 160 × 160 × 160, patches with batch size one. An additional four convolutional layers with kernel size three were added prior to the final 1×1×1 convolutional layer in the standard U-Net architecture as this was found to increase stability during training. All models were trained on the same 22 images while the remaining 10 images were equally split into validation and testing data. We further compare against the results that we reported in our preliminary work, where MultiRes could only be trained with single-channel *T*_1_--weighted input images.

We perform a similar experiment on the brain dataset comparing to an additional baselines, a multi-atlas propagation method, in order to evaluate the model’s extrapolation properties to other parts of the body. Similar to the whole-body dataset 30% of the brain data was excluded from training and split into validation and testing data.

## 3 Results

### 3.1 Quantitative evaluation

In order to assess the synthesis results quantitatively we calculate the Mean Squared Error $\left(MSE=\frac{\Sigma {\left(pCT-CT\right)}^{2}}{V}\right)$, where *V* denotes the total number of non-zero voxels) and Mean Absolute Error $\left(MAE=\frac{\Sigma \left|pCT-CT\right|}{V}\right)$ between the synthesised pCT and the ground truth CT (Table 1). It can be seen that the MultiRes method without uncertainty performs the best in terms of MAE averaged across all inference subjects. The lowest MSE is achieved by the MultiRes_*unc*_ network. A paired t-test was performed to show that the results of the MultiRes models are significantly better (p-value < 0.05) compared to the single resolution baseline methods. The MultiRes networks have a higher number of trainable variables than HighRes3DNet and DBR, however, the afforded performance increase compensates for this. Results from our preliminary work show that training MultiRes and MultiRes_*unc*_ with a single input channel leads to slightly superior results for MultiRes (MAE: 62.42 HU ± 6.8 HU; MSE: 11347.16 HU^2^ ± 3089.12 HU^2^) while the performance of MultiRes_*unc*_ is inferior (MAE: 80.14 HU ± 15.81 HU; MSE: 14113.83 HU^2^ ± 3668.79 HU^2^).

The results of the additional validation on the brain dataset are demonstrated in Table 2. It can be observed that all deep learning based methods outperform the more traditional multi-atlas propagation method. For the deep learning based methods, similar results are achieved compared to the results on the whole-body dataset. Both MultiRes models significantly outperform all baseline methods (p-value < 0.05) in MAE and MSE. When adding uncertainty to the MultiRes model, the performance is increased compared to its uncertainty unaware counterpart, suggesting that the network compensates for more uncertain regions during optimisation.

### 3.2 Qualitative evaluation

**Figure 2:**
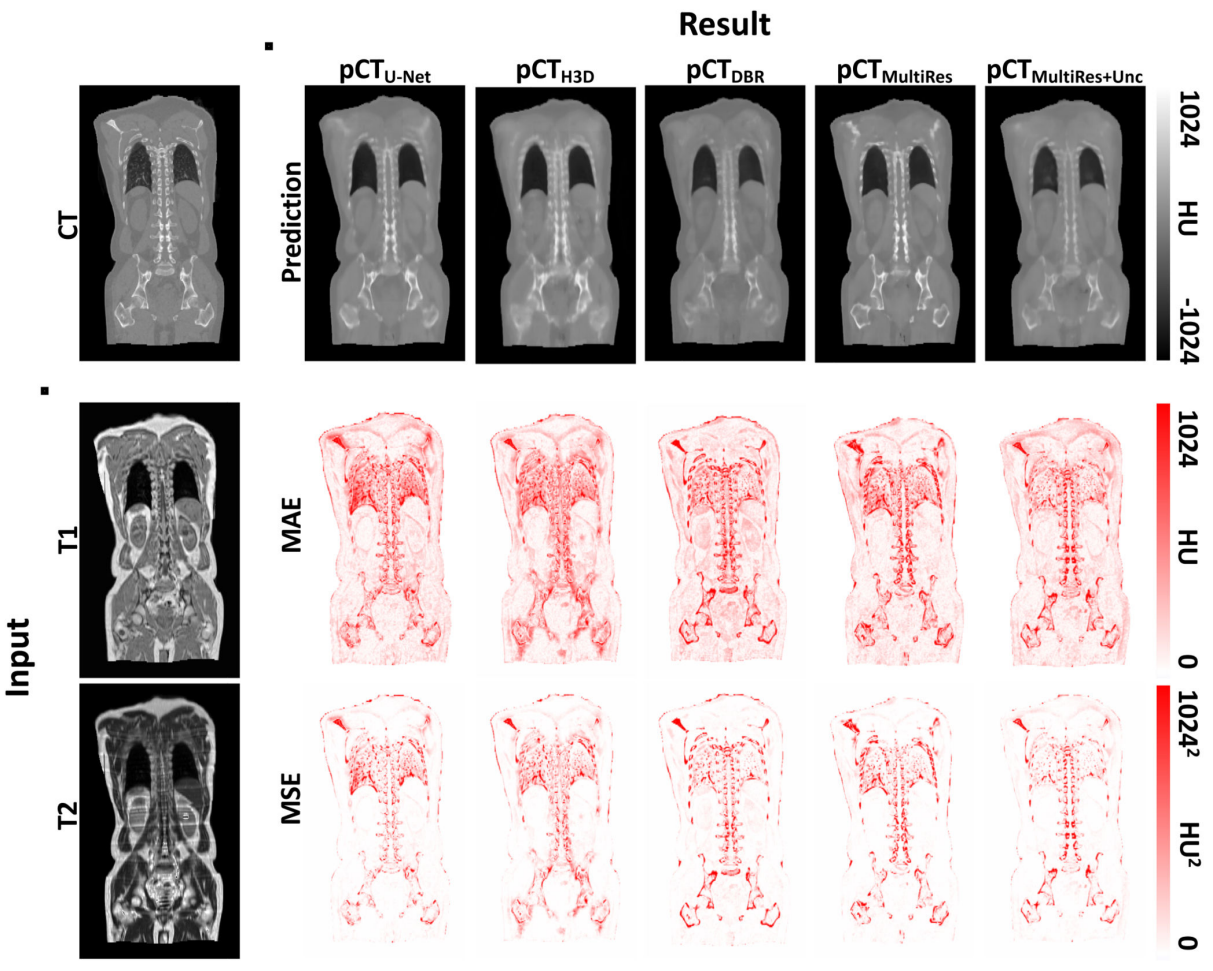
Ground truth CT and input *T*_1_- and *T*_2_-weighted MR images (first column) followed by predicted pseudo CT images with corresponding Mean Absolute Error (MAE) and Mean Squared Error (MSE) for 3D U-Net, HighRes3DNet, Deep Boosted Regression, MultiRes without uncertainty and MultiRes_*unc*_ including uncertainty estimation.

Fig. 2 shows the ground truth CT and the pCT predictions generated with 3D U-Net, HighRes3DNet, DBR, MultiRes and MultiRes_*unc*_ with uncertainty and the subject’s MR image as well as each model’s MAE and MSE.

The pCT images generated with 3D U-Net, HighRes3DNet and MultiRes appear sharp and exhibit high bone fidelity. DBR and the uncertainty aware MultiRes_*unc*_ seem blurrier with lower intensities in the bone. This is likely due to the network not being confident in predicting high bone intensities. This observation is confirmed by the MAE and MSE. The error suggests that the highest source of errors for all models stems from bones. 3D U-Net and HighRes3DNet also struggle to reconstruct lung intensities correctly. The lungs, having a significantly larger cross-sectional area, are visible, but lack internal consistency. Note, that all metrics used in this work are of absolute nature as opposed to relative, thus it is expected that errors in high intensity regions contribute most to the overall error.

The MultiRes model exhibits the highest bone fidelity; the individual vertebrae are clearer, with intensities more in line with what would be expected for such tissues, and the femurs have more well-defined borders. It is interesting to note that the MSE of the MultiRes_*unc*_ model is lower than the MSE of its uncertainty unaware counterpart. This shows that the majority of residuals in the pCT generated by MultiRes_*unc*_ are in a lower range than MultiRes without uncertainty and therefore when squared do not contribute as much to the MSE. This is likely because the network is less confident in bone regions, whereas the models that do not compensate for uncertainty are overly confident and predict high bone intensities in the wrong place, resulting in a high error.

**Figure 3:**
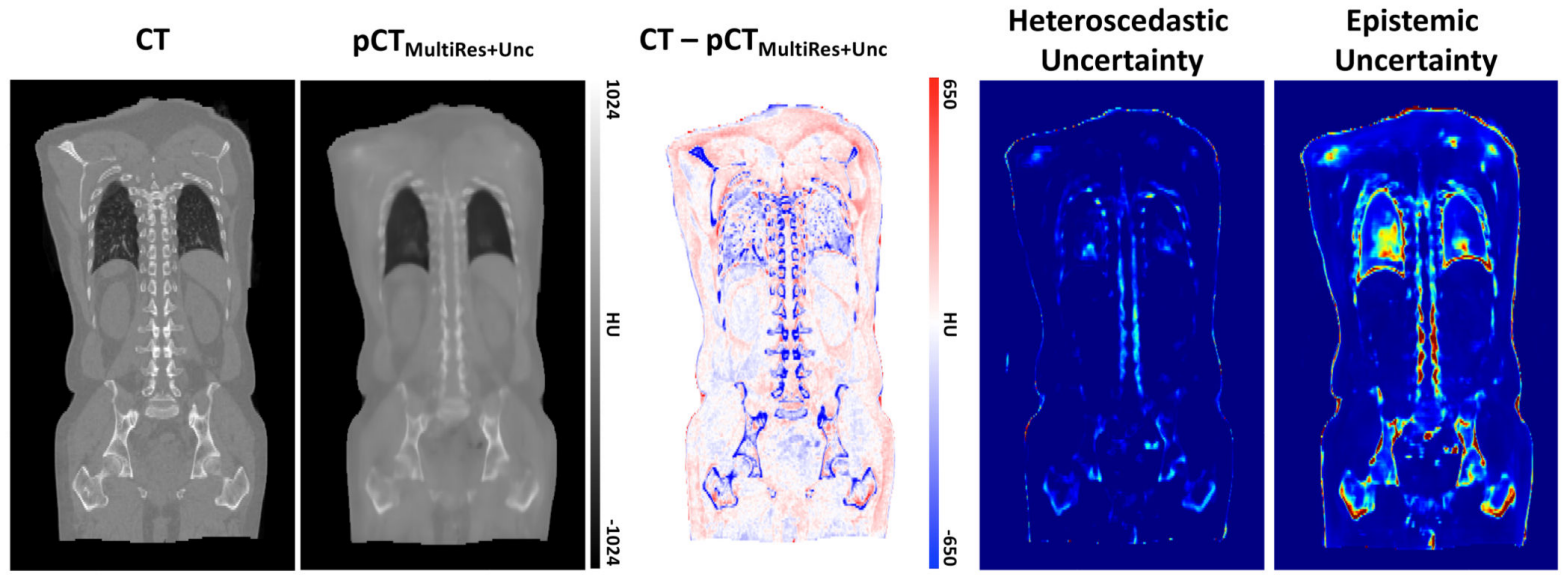
From left to right: Whole-body CT ground truth, pseudo CT prediction of MultiRes_*unc*_, corresponding residuals, heteroscedastic uncertainty and epistemic uncertainty. Both uncertainties correlate with the residual error map.

**Figure 4:**
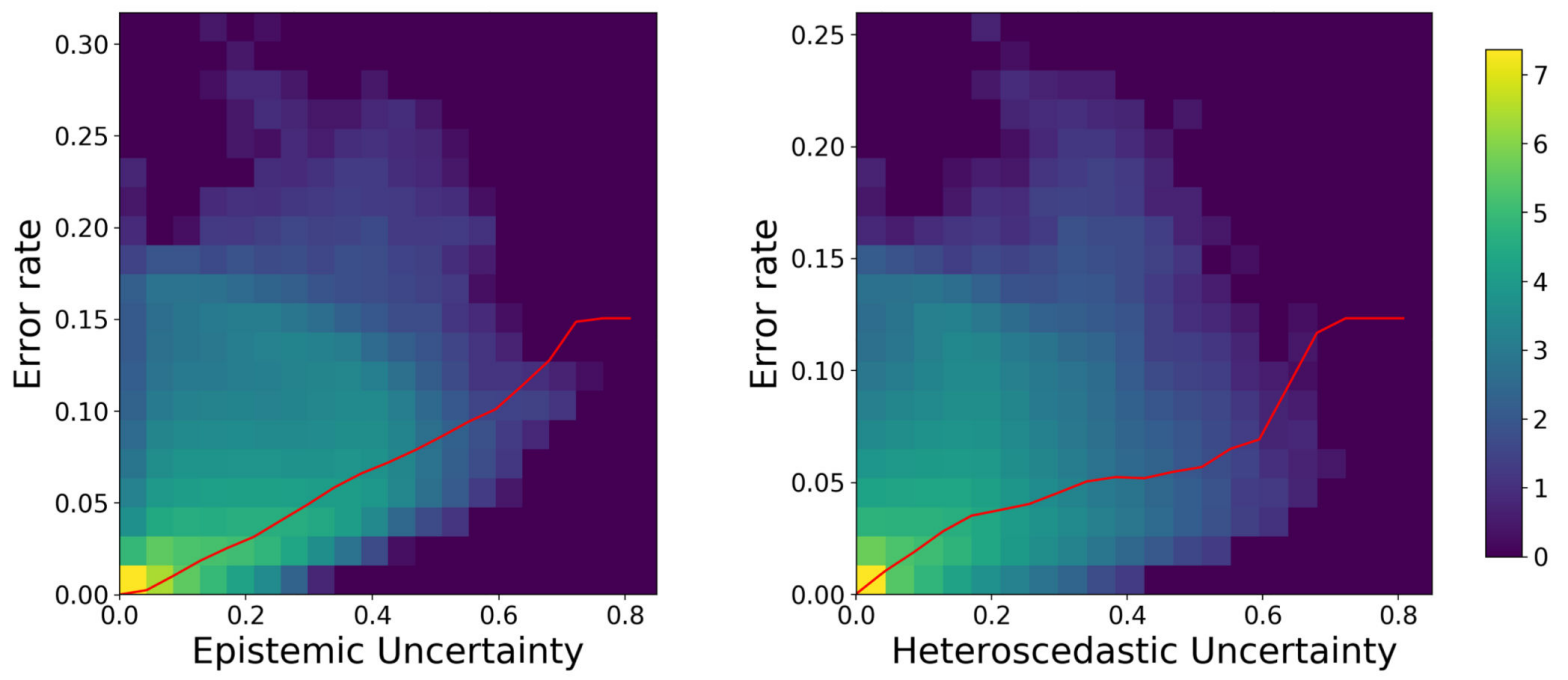
Joint histogram of prediction uncertainty and error rate for MultiRes_*unc*_ network: Epistemic (left), Heteroscedastic (right). Low correlation between uncertainty and error rate is shown in purple and high correlation is shown in yellow. The average error rate at different levels of uncertainty is depicted by the red line. The error rate tends to increase with a higher uncertainty, which shows that the network correlates uncertainty to regions of high error.

Figure 3 presents the benefits afforded to MultiRes_*unc*_ for being uncertainty aware. The joint histograms for epistemic uncertainty (left) and heteroscedastic uncertainty (right) are generated by calculating the error rate, taken as the difference between the ground truth CT and pCT averaged across N=50 dropout samples, at different levels of both epistemic and heteroscedastic uncertainty (standard deviations per voxel) and taking the base 10 log. The red line describes the average error rate at each level of uncertainty. It can be observed that a significant correlation between uncertainty and error rate exists, suggesting that the model appropriately assigns a higher uncertainty to those regions that are challenging to predict. This correlation is likewise observed when comparing the maps of epistemic and heteroscedastic uncertainty with the corresponding error map, which is demonstrated in Fig. 4. In areas around structure borders both epistemic and heteroscedastic uncertainties show large values, as expected. The borders between tissues are not sharp and there is, therefore, some ambiguity in these regions, which is mirrored by the corresponding overlapping error in the residuals. In general, a larger amount of data should diminish the epistemic uncertainty by providing the network with a greater number of samples from which the correspondence between MR and CT images can be learned within these areas. The observed blurriness, however, could result in some inconsistency in the synthesis process, which would still be captured by the heteroscedastic uncertainty.

We observed a high degree of uncertainty in the vicinity of air pockets. Unlike corporeal structures, it is expected that these pockets are subject to deformation between the MR and CT scanning sessions (and even between *T1* and *T2* acquisitions), which leads to a lack of correspondence between the acquisitions in these regions. This results in the network attempting to synthesise a morphologically different pocket to what is observed in the MR, resulting in a high degree of uncertainty.

**Figure 5:**
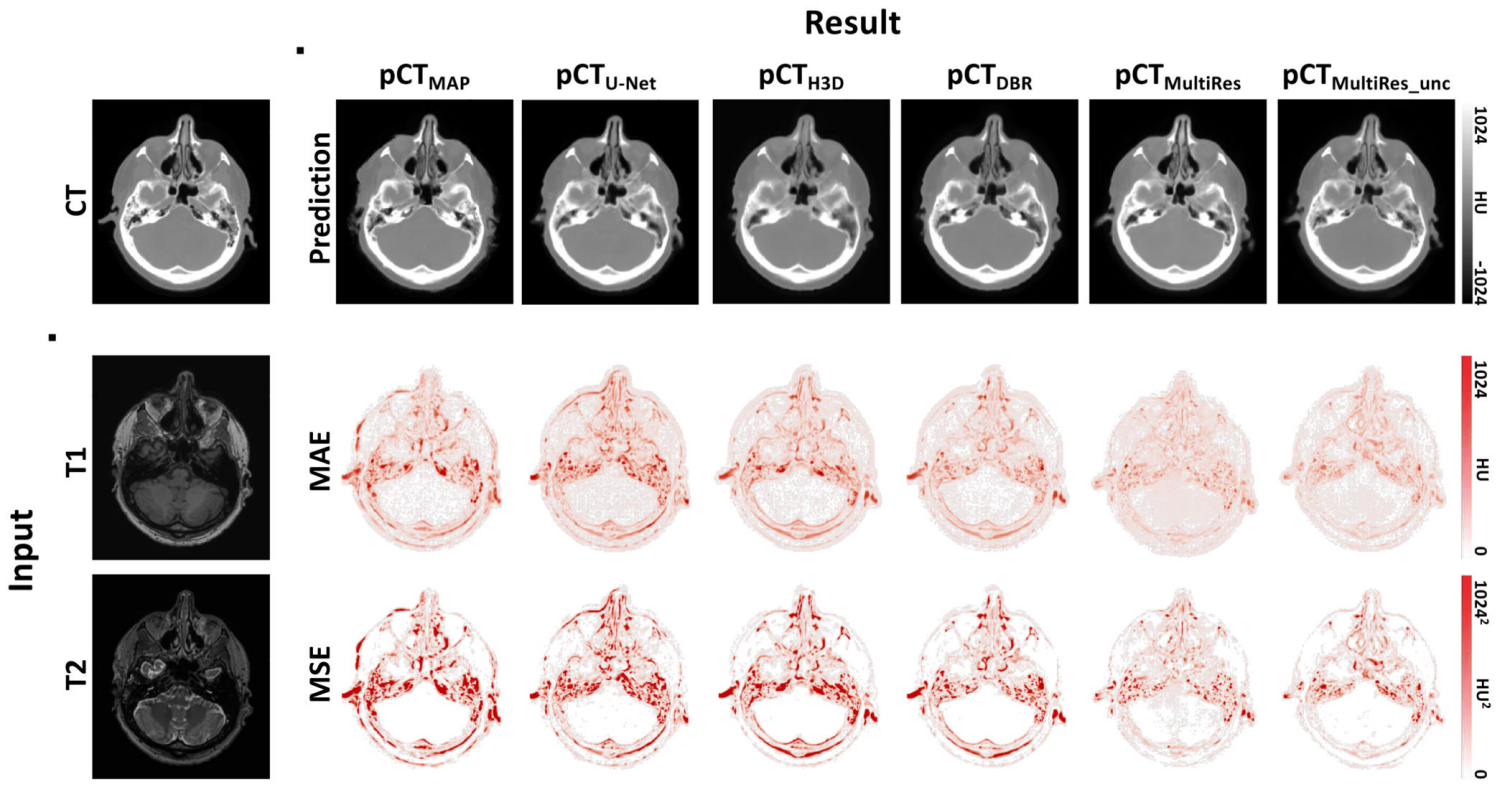
Ground truth CT and input *T*_1_- and *T*_2_-weighted MR images (first column) of the brain followed by predicted pseudo CT images with corresponding Mean Absolute Error (MAE) and Mean Squared Error (MSE) for a multi-atlas propagation method, 3D U-Net, HighRes3DNet, Deep Boosted Regression, MultiRes without uncertainty and MultiRes_*unc*_ including uncertainty estimation.

The additional validation results on the brain database can be seen in Fig. 5; the ground truth CT and the pCT predictions generated with a multi-atlas propagation method, 3D U-Net, HighRes3DNet, DBR, MultiRes and MultiRes_*unc*_ with uncertainty are shown alongside with the subject’s MR image as well as the models’ corresponding MAE and MSE. It can be seen that similar to the results on the whole-body dataset the biggest error source is bone. This is especially evident in the MSE of the multi-atlas propagation method, 3D U-Net, HighRes3DNet and DBR, where misclassified bone intensities are squared leading to a large error. However, both MultiRes models reconstruct sharp results and exhibit a lower residual error compared to all baseline methods. Only a small number of voxel intensities are wrongly classified resulting in an overall decreased error.

**Figure 6:**
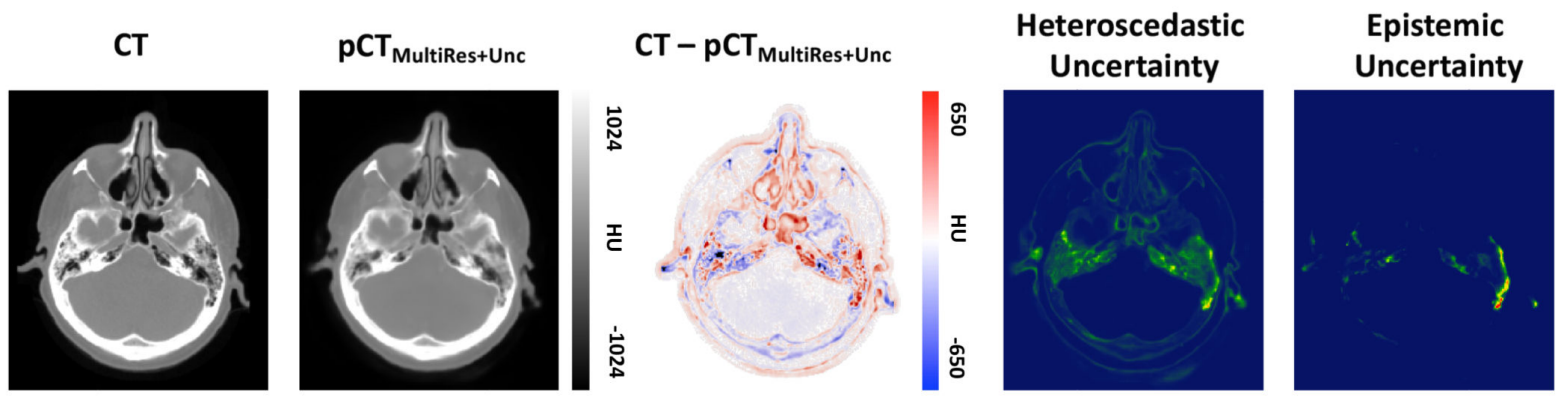
From left to right: Brain CT ground truth, pseudo CT prediction of MultiRes_*unc*_, corresponding residuals, heteroscedastic uncertainty and epistemic uncertainty. Both uncertainties correlate with the residual error map.

Figure 6 shows a pCT of the brain synthesised with MultiRes_*unc*_ and the correlation between the maps of epistemic and heteroscedastic uncertainty with the corresponding error map. Similarly to the whole-body experiment both epistemic and heteroscedastic uncertainties exhibit the largest values around bone and tissue boundaries, which is expected. The modelled heteroscedastic uncertainty is mainly evident in the cavity of the middle ear, which is a particularly difficult area to model for the network due to the small cartilage tissue structures. The epistemic uncertainty also shows a high value in the cavity of the middle ear as well as additional uncertainty at bone/tissue boundaries. However, the epistemic uncertainty is likely to diminish with a larger dataset.

## 4 Discussion and Conclusions

In this work we show the superior performance of MultiRes, a novel learning scheme for multi-channel multi-resolution image translation specifically aiming at MR to CT synthesis of the whole body, and MultiRes_*unc*_, an uncertainty aware version of this model that incorporates uncertainty as a safety measure and to account for intrinsic data noise. We showcase a significant performance increase (p-value < 0.05) of MultiRes and MultiRes_*unc*_ by comparing it to three single-resolution CNNs: 3D U-Net, HighRes3DNet and DBR. We further demonstrate the importance of modelling uncertainty, showing that MultiRes_*unc*_ can identify regions where the translation from MR to CT image is most difficult.

In a data-scarce environment, it becomes especially important to quantify uncertainty as networks are unlikely to have sufficient evidence to fully converge. After all, it is inevitable to accurately align CT and MR images when validating the voxel-wise performance of any image synthesis algorithm until other suitable methods have been established that allow us to validate the synthesis quality on non-registered data.

Although the synthesis results of MultiRes_*unc*_ seem slightly blurrier compared to MultiRes from a qualitative standpoint, we posit that the additional insight that is introduced by modelling uncertainty and the superior results on a quantitative basis can compensate for this. Compared to all baseline methods MultiRes and MultiRes_*unc*_ models do not tend to be overly confident, thus, they do not result in crucial bone misclassification.

While the model does not reconstruct bone-based structures as well as its uncertainty agnostic counterpart in the whole-body dataset, it still outperforms all baseline models.

Additionally, we tested the MultiRes network on a separate brain dataset. In general, synthesising CT images of the head is a much easier task due to the homogeneity of tissue intensities in the CT. Furthermore, the skull naturally exhibits a more symmetric anatomy compared to the whole body, making it easier for convolutional neural networks to learn the spatial image context. Lastly, images of the head are significantly smaller than whole-body images, thus patches include a larger field of view, which is one of the main challenges when working with whole-body images. The results show that the uncertainty aware MultiRes_*unc*_ network achieves the best synthesis results with a MAE of 57.01HU ± 17.96HU, suggesting its robustness and extrapolation properties to different datasets.

To summarise, we show that a multi-scale/resolution network, namely MultiRes, for MR to CT synthesis, outperforms single-resolution alternatives in whole-body and brain MR to CT synthesis applications. Furthermore, by incorporating epistemic uncertainty via test time dropout, and heteroscedastic uncertainty by casting the model probabilistically, we can showcase those regions that exhibit the greatest variability, which provides a measure of safety from an algorithmic point of view. We demonstrate that these regions have a high correlation with the residuals obtained by comparing the outputs with the ground truth, emphasising the importance of modelling uncertainty. We argue that the slight decrease in the MAE of the uncertainty aware model on the whole-body experiment is insignificant compared to the important additional information provided by the uncertainty. In future work we would like to explore how the uncertainty information could provide additional value when the pCT is used for PET/MR AC. This would allow us to reconstruct PET images including the voxelwise PET uncertainty of the uptake distribution, thus acting as a measure of PET reconstruction confidence. We further plan to perform a cross-validation study and test the model’s performance for out-of-distribution samples, e.g. when anatomical abnormalities such as tumors are present.

## Figures and Tables

**Table 1:** MAE and MSE across all experiments on the whole-body dataset including number of trainable variables. Bolded entries denote best model (p-value < 0.05).

Experiments	Model parameters	MAE (HU)	MSE (HU^2^)
3D U-Net	14.49M	92.89 ± 13.30	37358.07 ± 11266.56
HighRes3DNet	0.81M	89.05 ± 8.77	23346.09 ± 3828.22
DBR	1.62M	77.58 ± 3.20	19026.56 ± 2779.69
MultiRes	2.54M	**72.87** ± **2.33**	18532.23 ± 1538.41
MultiRes_*unc*_	2.61M	73.90 ± 6.24	**16007.56** ± **2164.76**

**Table 2:** MAE and MSE across all experiments on the brain dataset including number of trainable variables. Bolded entries denote best model (p-value < 0.05).

Experiments	Model parameters	MAE (HU)	MSE (HU^2^)
Multi-Atlas	N/A	132.15 ± 68.89	75364.30.07 *±* 62627.20
3D U-Net	14.49M	86.18 ± 9.95	21624.78 ± 6095.86
HighRes3DNet	0.81M	70.52 ± 10.80	19876.87 *±* 5804.39
DBR	1.62M	65.21 *±* 13.01	17308.84 *±* 6923.93
MultiRes	2.54M	57.52 ± 17.79	9611.25 ± 6251.68
MultiRes_*unc*_	2.61M	**57.01 *±* 17.96**	**7291.80 *±* 2857.76**

## Abbreviations

The following abbreviations are used in this manuscript:

AC: Attenuation Correction
ADAM: Adaptive moment estimation
CNN: Convolutional neural network
CT: Computed Tomography
DBR: Deep Boosted Regression
HU: Hounsfield unit
MRI: Magnetic Resonance Imaging
PET: Positron Emission Tomography
MAE: Mean absolute error
MSE: Mean squared error
MultiRes: Multi-resolution network
MultiRes_*unc*_: Uncertainty aware multi-resolution network
*μ*-map: Attenuation map
pCT: pseudo CT
*T*_1_: Spin-lattice relaxation time
*T*_2_: Spin-spin relaxation time
